# 

**Published:** 1895-06

**Authors:** 


### Communicated.

THE AMERICAN DENTAL ASSOCIATION.—The American Dental Association holds its next meeting at Asbury Park, N. J., Tuesday, August 6th, 1895. I am requested by the President, Dr. J. Y. Crawford, to give notice that it is the privilege and duty of each State and Local Society to appoint and send delegates to the American Association.

Each State and Local Society which has adopted substantially the same Code of Ethics as that governing the conduct of members of the American Dental Association is entitled to one representative for every five members and fractional part thereof.

Blank certificates for delegates may be had on application to the Corresponding Secretary.

Many questions of interest will come up for discussion at this meeting, and every Dental Society in the United States should be fully represented in this, our National Convention.

EMMA EAMES CHASE,
Corresponding Secretary.

### ILLINOIS STATE DENTAL SOCIETY.

The thirty-first annual meeting of the Illinois State Dental Society was held at Galesburg, May 14 to 17, 1895. About 200 were in attendance. The following named persons were elected officers for the ensuing year: President, Walter A. Stevens: of Chicago; Vice-president, C. R. Taylor of Streator; Secretary, Louis Ottofy of Chicago; Treasurer, Edgar D. Swain of Chicago; Librarian, J. R. Rayburn of Fairbury; Chairman of Executive Committee, W. A. Johnston of Peoria.

The next meeting will be held at Springfield, May 12 to 15, 1896.

LOUIS OTTOFY, Secretary,
Masonic Temple, Chicago.

---

### NATIONAL ASSOCIATION OF DENTAL FACULTIES.

The annual meeting of this Body will be held at Asbury Park, N. J. on Saturday August 3d at 10 o'clock a. m. It is very desirable that all the Colleges having membership be promptly present at that hour, as much important business will be before the Association, and the time allotted is usually short for the work to be done.

The executive Committee of the Association will meet on Friday previous at 10 o'clock at the same place. All business for that committee should, so far as possible be in their hands before the meeting in order that there be no delay.

J. TAFT, Chairman Ex. Com.

LOUIS OTTOFY, Secretary.
Masonic Temple, Chicago.

### THE NATIONAL ASSOCIATION OF DENTAL EXAMINERS.

The annual meeting will be held in the Parlors of the "Hotel Columbia" Asbury Park, N. J., on Monday August 5th, at 10 a. m., and at other times during the week as becomes necessary between the sessions of the American Dental Association. It is important that every State Board be represented. Applications from Boards not in Membership will recieve immediate attention.

CHAS. A. MEEKER, D. D. S., Secretary.
29 Fulton St., Newark, N. J.

### NEW JERSEY STATE DENTAL SOCIETY.

The twenty-fifth annual meeting (The Silver Anniversary) of the New Jersey State Dental Society will be held in the "Auditorium" Asbury Park, commencing August 1st, at 10 a. m. and continuing through Friday, Saturday and Monday a. m., closing in time for the meeting of the American Dental Association commencing Tuesday August 6th. at 10 a. m.

The "Auditorium" is the Ideal place for a summer dental meeting being situated in the Middle of an entire block fronting the surf, with large windows opening from every side in one continuous row, thirty large windows with North light for clinics and 390 x 25 feet for exhibits.

A branch of the Asbury Park Post Office will be established in the "Auditorium" and a bureau for general information with attendants constantly on hand.

The New Jersey headquarters will be in the "Hotel Columbia" with rates from $2.00 to $3.00 per day, several large Hotels have made contracts from $2.50 to $4.00 per day and smaller Hotels from $8.00 to $12 per week board.

Full particulars and rates with map of Asbury Park and a plan of the "Auditorium" will appear in the program.

CHAS. A. MEEKER, D. D. S., Secretary,
29 Fulton St., Newark, N. J.

